# Characterization of root-associated bacteria from paddy and its growth-promotion efficacy

**DOI:** 10.1007/s13205-013-0158-9

**Published:** 2013-07-31

**Authors:** Yachana Jha, R. B. Subramanian

**Affiliations:** 1 N. V. Patel College of Pure and Applied Sciences, Sardar Patel University, V. V. Nagar, Anand, Gujarat India; 2 BRD School of Biosciences, Sardar Patel University, Post Box No. 39, V. V. Nagar, Anand, 388120 Gujarat India

**Keywords:** PGPR, Phosphate solubilization, ACC deaminase, Phytohormones, Siderophore, NifH gene

## Abstract

Bacteria from rhizosphere (*Bacillus pumilus*) and endorhizophere (*Pseudomonas pseudoalcaligenes*) of rice plant were isolated and evaluated for their effect on the growth-promotion efficiency on rice in greenhouse. Ability to solubilize phosphate, siderophore, indoleacetic acid (IAA), gibberellin production and utilization of ACC (1-aminocyclopropane-1-carboxylate) as sole nitrogen source were evaluated, which were produced in high concentration by *P. pseudoalcaligenes* in this present study. Inoculation of isolated microorganism resulted in the reduction of pH (from neutral to acidic) of the medium used for phosphate solubilization, and has direct relation with titratable acidity, but gluconate production showed an opposite trend. *P. pseudoalcaligenes* better helped the plant to overcome or suppress fungal pathogen infection by producing β-1, 3-glucanase and chitinase as well as also have enhanced dry weight, plant height, and root length. Based on these results, *P. pseudoalcaligenes* in this study proved a better candidature as PGPR than *B. pumilus*.

## Introduction

Rice (*Oryza sativa*) is one of the most important stable food crops in the world. In Asia, more than two billion people get 60–70 % of their energy requirement from rice and its derived products. To sustain present food self-sufficiency and to meet future food requirements, there is a need to increase rice productivity by 3 % per annum (Thiyagarajan and Selvaraju 2001). However, the production of rice is adversely affected by a number of biotic (viruses, bacteria, fungi, nematodes, insects, etc.) and abiotic (unfavorable soil, wound, temperature, flooding, etc.) stresses (Goff 1999). The techniques such as use of resistant variety, crop rotation, chemical method, and several other control methods have been used to meet the requirement of growing population. But these techniques have several drawbacks. Chemical methods have been used since long but they damage the natural beneficial insects, environment and also contaminate the natural resources. So presence of plant growth-promoting N_2_-fixing bacteria and the possibility of a significant increase in plant performance and yield under nutrient limiting conditions have been discussed for many years. In the context of increasing international concern for food and environmental quality, the use of PGPR for reducing chemical inputs in agriculture is potentially important. PGPR has been applied to crops in various forms to enhance growth, seed emergence and crop yield (Minorsky 2008). Although plants are naturally exposed to several phytopathogenic microorganisms, they exhibit tolerance to these pathogens, through various morphological, anatomical structures (cuticles, trichomes, stomata and tyloses) and biochemical mechanisms (such as phenols, phytoalexins, cyanogenic glycosides, protease inhibitors and hydrolases) (Caramori et al. 2004). The objective of present study is to characterize and elucidate the effect of isolated PGPR on plant growth promotion by producing phytohormones, siderophores, ACC (1-aminocyclopropane-1-carboxylic acid) deaminase, amplification of nifH gene and pathogenesis-related proteins (PR proteins).

## Materials and methods

### Characterization of plant growth-promoting mechanism

*Bacillus pumilus* and *Pseudomonas pseudoalcaligenes* strains were isolated from the rice field and identified (data not shown) as per our published method (Jha et al. 2011). Their growth-promotion efficiency was analyzed by their ability to solubilize phosphate, produce siderophore, indoleacetic acid (IAA), gibberellins, and utilizes ACC as sole nitrogen source and to overcome or suppress infection by producing β-1, 3-glucanase and chitinase.

### Quantitative estimation of phosphate solubilization

Phosphate solubilization was estimated by Ames (1964) method by inoculating fresh culture in freshly prepared 10 % ascorbic acid mixed with cold 0.42 % ammonium molybdate in 1 N H_2_SO_4_ in a ratio of 1:6 and incubated on an ice bath for at least 1 h. The readings were taken at an interval of 3 days in 3 replicates.

### Estimation of titratable acidity and gluconic acid production

Titratable acidity was determined by titrating 1 ml of culture filtrate against 10 mM NaOH in presence of phenolphthalein (Whitelaw et al. 1999). For estimation of gluconic acid released by cultures, 1 ml of culture supernatant was used and estimation was done by Welcher’s method (1958). The result was expressed in mmol l^−1^ and carried in 3 replicates.

### Estimation of Indole acetic acid and gibberellic acid production

Overnight grown cultures were inoculated in N-broth containing 0.2 % yeast extract, 1 % glucose and incubated for 24 h, and indole acetic acid was estimated by Gordon and Weber (1951) method. Gibberellic acid production was estimated by colorimetric method of Hohlbrook et al. (1961). Absorbance was measured at 254 nm and experiment was carried out in 3 replicates.

### Estimation of β-1, 3-glucanase and chitinase

Bacterial cultures were inoculated in N-broth and allowed to grow for 24 h at 30 °C on shaker at 150 rpm. The bacterial culture was centrifuged at 10,000*g* for 20 min and the supernatant was used as enzyme source. β-1, 3-glucanase activity expressed as nmol min^−1^ mg^−1^ was estimated by method of Pan et al. (1991). Chitinase activity was estimated by Reissig et al. (1995) method and expressed as μmol Glc-NAc equivalents’ s^−1^ g^−1^. Experiment was carried out in 3 replicates.

### Estimation of hydroxymate and catechol siderophores production

Estimation of hydroxymate type siderophores was carried out by Mayer and Abdallah’s (1978) method and catechol groups was estimated by Arnow’s (1937) colorimetric assay method.

### ACC deaminase activity assay

ACC deaminase activity of bacterial isolates was estimated by Penrose et al. (2001) method, and the amount of F-ketobutyric acid (F-KA) generated from the cleavage of ACC was monitored using spectrophotometer. The amount of F-KA produced during this reaction was determined by comparing the absorbance at 540 nm of a sample to a standard curve of F-ketobutyrate and expressed as the amount of F-ketobutarate produced per mg of protein per hour.

### Extraction of genomic DNA and PCR amplification of nifH gene

For DNA extraction, colonies from bacterial isolates were cultured in 3 ml of liquid 1/2 DYGS medium overnight at 30 °C. The cells were centrifuged and further used for DNA extraction. Genomic DNA was extracted and purified by use of the Fast DNA spin kit (Qbiogene Inc., CA, USA) according to the manufacturer’s protocol. Amplification of the nifH gene from the extracted DNA was performed using the primers Pol F (5′-TGCGAYCCSAARGCBGACTC-3′) and Pol R (5′-ATSGCCATCATYTCRCCGGA-3′). Amplification was performed in 50 ml final volume containing 1 ml genomic DNA (50 ng), 20 pmol each of forward and reverse primer, PolF and PolR, a 200 mM concentration of each of dNTPs (Sigma, USA), 10XTaq polymerase buffer and 2.5 U of Taq polymerase (Sigma, USA). PCR conditions consisted of initial denaturation step at 94 °C for 4 min, 30 amplification cycles of denaturation at 94 °C for 1 min, annealing at 55 °C for 1 min and primer extension at 72 °C for 2 min; followed by a final extension at 72 °C for 5 min with MyCycler™ PCR System (BioRad, USA). Aliquots of the PCR products were analyzed in 1.5 % (w/v) agarose gels (Sigma, USA) by horizontal gel electrophoresis. PCR products were eluted from agarose gel, purified and sequenced.

### Inocula preparation, seedling germination and greenhouse study

Bacteria were grown in yeast mannitol broth (YMB) and exponentially growing cells in shaken broth culture were used for inoculation. Rice seeds were surface sterilized by 70 % ethanol in a flask and were treated with 1 % sodium hypochlorite for 2 min followed by six times washing with sterile water. After that, the seeds were soaked in various PGPR broths. Seeds soaked in normal broth were treated as control. Seeds of both inoculated and controls were put in sterilized petri dishes containing filter paper (Whatman #102) and the petri dishes were kept in an incubator at 30 °C for 120 h. After soaking, the air-dried seeds were used for germination and the survival percent.

The bacterial isolates, either alone or as a mixture, were assessed for their efficiency in suppressing rice blast under greenhouse conditions. The spore suspension of *Magnaporthe grisea* with a spore load of 10^4^ conidia ml^−1^ was sprayed on the plants, which caused more than 75 % infection under greenhouse conditions. Observations on the percent disease incidence of rice blast were recorded. Disease index was calculated as grades 0–5 by Sriram et al. (1999) method using the formula:

Disease index=Total grade×100/No.of sheaths observed×maximum grade.

Plant obtained from germinated seeds were transferred to plastic pots containing sterilized sand-perlite (1:1) and kept in a greenhouse. The plants were irrigated with water and Hoagland nutrient solution once a week. Shoots’ and roots’ lengths, fresh and dry weights were determined after 4 weeks. All experiments were carried in 3 replicates.

### Statistical analysis

Data were analyzed by one-way ANOVA (analysis of variance). All treatments were replicated 3 times. Differences were considered to be significant at the *P* < 0.05 level. Means were compared by Fisher’s protected LSD.

## Results and discussion

Rhizosphere is the most dynamic ecological niche where inter and intra species interactions of microbes, such as bacteria, fungi and protozoa, occur due to the presence of a rich and diverse microbial food source (Bais et al. 2006). The importance of rhizosphere microbial populations for maintenance of root health by nutrient uptake, and tolerance of environmental stress are now well-recognized (Bowen and Rovira 1991). Secondly, biofertilization by PGPR improve nutrient status of plant by associative nitrogen fixation, phosphorus solubilisation and siderophores production, altering the permeability and transforming nutrients in the rhizosphere thus increasing their bio-availability (Mantelin and Touraine 2004). In addition, hormonal effects occur when PGPR either produce or metabolize chemical signaling compounds that directly impact on plant growth and function (Patten and Glick 2002).

In the present study, two bacterial isolates *P. pseudoalcaligenes* and *B. pumilus* were selected from thirty-five isolates obtained from the paddy field at the botanical garden of S. P. University, Gujarat, India and were found to be efficient with reference to their phosphate solubilizing capability. Bacterial genera such as *Bacillus, Pseudomonas* and *Brevibacillus* are known to be promoting growth, and yield in different non-leguminous plants was also reported by Selva kumar et al. (2008). Phosphorus is one of the major nutrients, second only to nitrogen in requirement for plants. Most of phosphorus in soil is present in the form of insoluble phosphates and cannot be utilized by the plants (Pradhan and Sukla 2005). In the present study, the phosphate released by *B. pumilus* was increased by 5 times and titratable acidity by 1.5 times after 9 days of inoculation, while phosphate by *P. pseudoalcaligenes* released was increased by 13 times and titratable acidity by 4.1 times after 12 days of inoculation in the medium. Both the isolates were able to solubilize phosphorus with the production of gluconic acid as shown in Table 1. Zaidi et al. (2009) reported that the mineral phosphorus solubilization could probably be due to secretion of organic acids, such as gluconic, 2-ketogluconic. Production of organic acids for solubilisation of phosphates is a very well-known mechanism (Jones 1998); the reason for reduction in pH in present study may also be due to production of organic acids gluconate by the isolates. The reduction in pH also has role in the phosphate solubilization and has been supported by Stumn and Morgan (1996), who reported that below pH 5, solubilization of phosphates of Ca, Al and Fe(III) increase.

**Table 1:** Phosphate released, titratable acidity and gluconic acid concentration during solubilisation of tricalcium phosphate over incubation period of 12 days by the *P. pseudoalcaligenes* (*n* = 3)

Days	pH	Phosphate released (μg P ml^−1^)	Titratable acidity (×10^−2^)	Gluconate (×10^−4^ g %)
*P. pseudoalcaligenes*				
0	7.00 ± 0.01	70.50 ± 2.22	0	0
3	5.03 ± 0.03	225.00 ± 5.63	7.69 ± 0.10	6.42 ± 1.29
7	3.51 ± 0.01	565.50 ± 10.67	26.40 ± 0.12	4.68 ± 1.29
9	3.80 ± 0.05	726.50 ± 19.30	30.70 ± 0.10	3.62 ± 1.90
12	3.90 ± 0.05	934 ± 20.14.00	32.50 ± 0.13	2.20 ± 2.24
*B. pumilus*				
0	7.00 ± 0.05	72.5 ± 06.24	0	0
3	5.00 ± 0.03	211.5 ± 10.10	12.3 ± 0.15	10.20 ± 1.76
7	5.03 ± 0.01	242.5 ± 14.00	14.3 ± 0.30	10.20 ± 2.29
9	5.55 ± 0.03	385.0 ± 07.50	19.0 ± 0.10	8.92 ± 1.40
12	5.83 ± 0.02	182.5 ± 17.55	09.1 ± 0.10	6.72 ± 1.15

Values are mean of three replications. (*P* ≤ 0.05; LSD test)

**Fig. 1:**
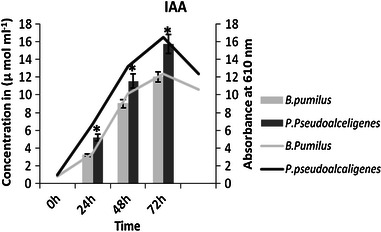
IAA production by the isolates at different time interval on suitable medium (*n* = 3) in *Bar graph* and growth curve of isolates in *Line graph*

**Fig. 2:**
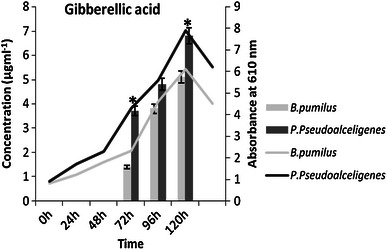
GA_3_ production by *B. pumilus* and *P. pseudoalcaligenes* at different time interval on suitable medium (*n* = 3) in *Bar graph* and growth curve of isolates in *Line graph*

Auxins and gibberelline may function as an important signal molecule in the regulation of plants growth and development. In this study, IAA and gibberellic acid production increased with time by both the isolates, production of IAA increased 4 times by *B. pumilus* and 3 times by *P. pseudoalcaligenes* (Fig. 1), while gibberellic acid increased 3 times by *B. pumilus* and only 2 times by *P. pseudoalcaligenes* in 96 h compared to initial concentration at 72 h (Fig. 2).

Siderophores are low molecular weight chemical compounds that scavenge iron, present in the environment as complexes and make this element available to the microorganisms (Neilands and Nakamura 1991). In the present study, the cultures were characterized for production of catechol and hydroxymate siderophores. *B. pumilus* produced 1.4 times higher catechol siderophore (12.9 μg ml^−1^) and production of hydroxymate siderophore was 1.3 times higher by *P. pseudoalcaligenes* (3.75 μg ml^−1^). These characters indicate that both isolates are better candidates for biofertilizer.

**Fig. 3:**
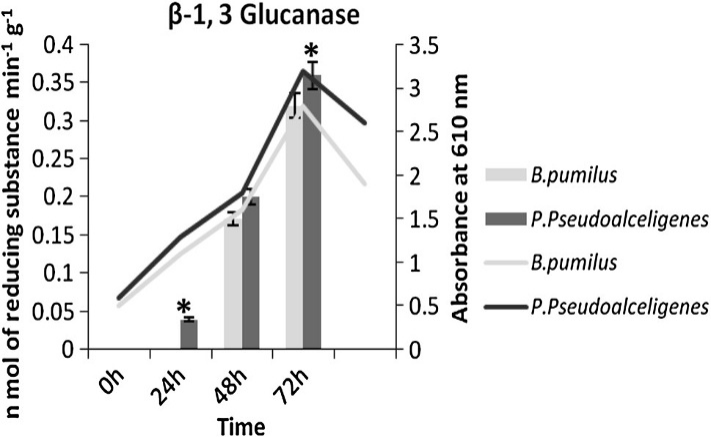
β-1,3 glucanase production by *B. pumilus* and *P. pseudoalcaligenes* at different time interval on suitable medium (*n* = 3) in *Bar graph* and growth curve of isolates in *Line graph*

Many microorganisms produce and release lytic enzymes that can hydrolyze a wide variety of polymeric compounds, including chitin, proteins, cellulose, hemicellulose and expression of these enzymes by different microbes which can sometimes result in the suppression of plant pathogen activities directly; β-1, 3-glucanase and chitinase contribute significantly to biocontrol activities (Palumbo et al. 2006). Chitin is the most frequently occurring structural element of many invertebrates and fungi, and chitinase enzyme attacks chitin polymer. The β-1, 3-glucanase and chitinase production increased in both the isolates with time duration. β-1, 3-glucanase was 5 times high in 48 h and 9 times high at 72 h by *P. pseudoalcaligenes,* while in *B. pumilus*, it only increased by 0.5 times in same time duration (Fig. 3). Chitinase increased 17 times by *B. pumilus* at third day of inoculation and 5 times high at 24 h of incubation by *P. pseudoalcaligenes*. Its production increased by 15 times by *B. pumilus* and 3 times by *P. pseudoalcaligenes* in a 3-day time duration (data already communicated). Microorganisms, which secrete a complex of mycolytic enzymes, are considered to be potential biological control agents of plants diseases Dal Soglio et al. (1998).

**Fig. 4:**
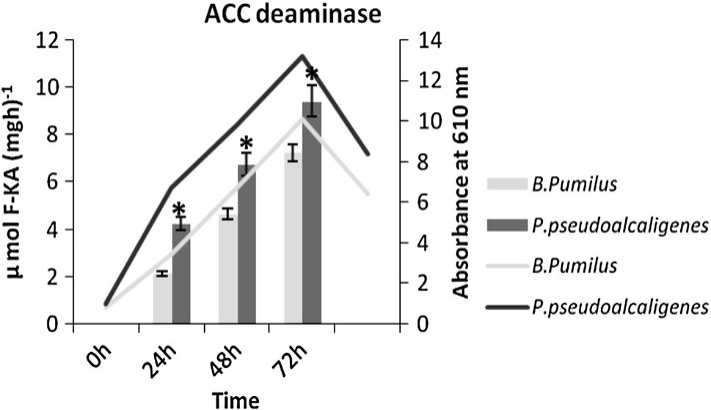
ACC deaminase production by *B. pumilus* and *P. pseudoalcaligenes* at different time interval on suitable medium (*n* = 3) in *Bar graph* and growth curve of isolates in *Line graph*

In the present study, the ACC deaminase activity also increased with time duration by both the isolates. It increased 3.4 times by *B. pumilus* and 2 times by *P. pseudoalcaligenes* after 72 h (Fig. 4). Belimov et al. (2002) also reported that *B. pumilus* and *Pseudomonas putida* showed ACC deaminase from the rhizoplane of pea (*Pisum sativum*) and Indian mustard (*Brassica juncea*).

**Fig. 5:**
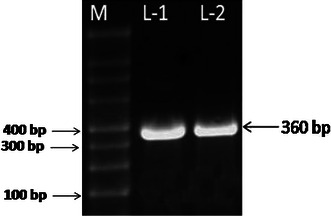
Agarose gel electrophoresis of amplified NifH gene of isolates. *M* marker 100 bp DNA ladder, *L-1* and *L-2* NifH gene from *B. pumilus* and *P. pseudoalcaligenes*, respectively

Presence of nifH gene was confirm by amplification of the structural gene for nitrogenase reductase (nifH) from the isolates, showed its potential for nitrogen fixation (Fig. 5). Xie et al. (2006) supported identification of nifH gene in the bacterial isolates from the rice field. In the greenhouse study the rice plants inoculated with isolates showed significantly higher plant height, root length and dry weight and also positive response on germination and survival percentage as shown in Table 2, and similar findings are reported by Chi et al. (2005). The present study strongly supports the development of biocontrol strategies using bacterial strains having antagonistic metabolites, to reduce the damage caused by plant pathogens. Present study showed that plants co-inoculated with PGPR and fungus *M. grisea* have disease index 38–43 % only in comparison to non-inoculated plants, where infection with fungus has 76 % disease index. The findings are supported by Ramamoorthy et al. (2002) who reported that PGPR plays a vital role in the management of various fungal diseases and Adesemoye et al. (2008) confirmed growth promotion by one representative each from both species of bacteria (*Pseudomonas* and *Bacillus*), but little variations were observed in bacterial effectiveness among parameters and crop types.

**Table 2:** Effect of PGPR on growth parameter under glasshouse study (*n* = 3)

Mean ± SD			
	Control	Control + *B. pumilus*	Control + *P. pseudoalcaligenes*
Germination (%)	61.1 ± 0.01	65.4 ± 0.01	71.8 ± 0.04
Survival (%)	85.7 ± 0.11	89.1 ± 0.03	91.3 ± 0.02
Plant height (cm)	11.1 ± 0.04	13.6 ± 0.04	16.3 ± 0.01
Root length (cm)	2.7 ± 0.03	3.1 ± 0.01	4.0 ± 0.04
Dry weight (cm)	0.5 ± 0.02	0.7 ± 0.11	0.9 ± 0.02

Data are represented as means per pot from three pot replicates, each containing 15 transplanted plants per strain (*P* ≤ 0.05; LSD test)

Observations were also supported by our studies on induction of defense related enzymes (Jha and Subramanian 2011) and accumulation of osmoprotectants (Jha et al. 2011) in presence of *P. pseudoalcaligenes* and *B. pumilus* alone and in combination helps the paddy under stress.
